# 

**Published:** 1882-01-20

**Authors:** 


### TABLE OF CONTENTS.

ORIGINAL AND SELECTED ARTICLES.

Two cases of Lithotomy. by Alexander F. Durham, M. D., of Georgia, 1. Something New—Nitric Acid a Remedy for Stings and Poisons, by A. L. Barry, M. D., o Georgia, 2. Recurrent Malarial Attacks, by R. L. Hinton, M. D., of Arkansas, 3. Emphysema, by R. L. Hinton, M. D., of Arkansas, 4. Use of Plaster-of-Paris in the Treatment of Club-Foot, by P. S. Connor, M. D., of Chicago, 5. An Old Doctor's Story, 7. A Case of Opium Habit of Six or Eight Years' Standing Treated Successfully with the Solid Extract of Coca, 9. Animal Vaccination, by William B. Carpenter, M. D., of London, England, 10. Self-Abortion, by Wm. H. Harrison, M. D., 11. The Sixteen Commandments of the Paris Academy of Medicine. Translated by D. C. Holliday, of New Orleans, 13. My Experience in Stammering and its Cure, by Thomas S. Gallaher, M. D., 15. Report on the Progress of Dentistry, by T. A. Chandler, M. D., 17.

ABSTRACTS AND GLEANINGS.

Notes on the Preparation and Properties of some New Remedies, 19. Cold Water Enemata in Diarrhœa of Children, 23. Explosive Mixtures, 24. Is there a Specific Urethritis? 25. Select Formulæ for Ingrowing Toe-Nails, 26. Supra-Pubic Operation for Lithotomy; Temperature of Sleeping-Rooms, 27. The Bacillus Lepræ; What Constitutes a Good Physician, 28. Black Sheep in the Flock; Treatment of Spermatorrhœa; What is Malaria?; New Hypodermic Syringe, 29 Bromide Ammonia in Cholera; Collyrium for Dissolving Metallic Foreign Bodies from the Cornea; Dyspepsia Caused by Tight Lacing; Improvement in Hypodermic Injection, 30.

SCIENTIFIC ITEMS.

Is there Life in Other Planets; To Hasten the Germination of Cotton Seed; To Preserve Iron from Rust, 31. Tongues and Gizzards; A New Gaseous Disinfectant; What is Sulphur; Cosmical Hail, 32.

PRACTICAL NOTES AND FORMULÆ.

Absorbent Cotton; Pruritus Vulvæ, 33. For Itching; Injection Brou; The Bromides; Formulæ Used in the New York Hospital—For External Use, 34.

EDITORIALS AND MISCELLANEOUS.

Our Journal for 1882; Brearley's Systematizer; Parke, Davis & Co.; Allen & Masser; J. Bradfield; Kidder & Laird, 36. Association of American Medical Colleges, 37. Pamphlets—Spiritualism at the Church Congress; Something Novel, Simple Economical and Complete; Vick's Floral Guide; Herman, Sibley & Co., 38. The Southern World; The Daily Florida Union; Book Notices, 39 Receipts; Special Notices, 40.

